# Hybrid approaches to allied health services for children and young people: a scoping review

**DOI:** 10.1186/s12984-024-01401-1

**Published:** 2024-07-19

**Authors:** Tal Krasovsky, Patrice L. Weiss, Liat Gafni-Lachter, Rachel Kizony, Naomi Gefen

**Affiliations:** 1 Department of Physical Therapy, Faculty of Social Welfare and Health Sciences, University of Haifa, 199 Abba Hushi Avenue, Haifa, 3498838 Israel; 2 Department of Pediatric Rehabilitation, The Edmond & Lily Safra Children’s Hospital, Sheba Medical Center, Ramat Gan, Israel; 3 Department of Occupational Therapy, Faculty of Social Welfare and Health Sciences, University of Haifa, Haifa, Israel; 4 The Helmsley Pediatric & Adolescent Rehabilitation Research Center, ALYN Hospital, Jerusalem, Israel; 5 College of Health and Rehabilitation Sciences, Department of Occupational Therapy, Sargent College, Boston University, Boston, USA; 6 Department of Occupational Therapy, Sheba Medical Center, Ramat Gan, Israel; 7 School of Occupational Therapy, Hebrew University, Jerusalem, Israel; 8 ALYN Hospital, Jerusalem, Israel

**Keywords:** Rehabilitation, Blended, Technology, Remote, eHealth, Hybrid, Telehealth

## Abstract

**Background:**

Hybrid models that integrate both in-person and remote health services are increasingly recognized as a promising approach. Nevertheless, research that defines and characterizes these models in children and young people is scarce and essential for establishing guidelines for implementation of hybrid allied health services. This scoping review evaluates four key aspects of hybrid allied health services in children and young people: 1. definitions, 2. service characteristics, 3. outcome measures, and 4. results of hybrid allied health services.

**Methods:**

Six databases were searched: Medline (Ovid), Embase, CINHAL, Psycinfo, Cochrane CENTRAL, and Web of Science. Of the 9,868 studies potentially meeting the inclusion criteria, 49 studies focused on children and young people. Following full-text review, *n* = 21 studies were included.

**Results:**

Terminology used for hybrid allied health services varied across studies which targeted diverse clinical populations and varied in study design, type and frequency of remote and in-person treatments. Over 75% of cases used custom-written software, limiting scalability. All interventions started in-person, possibly to establish a therapeutic alliance and solve technological issues. Most hybrid allied health services (67%) were in mental health, while only a minority involved physical, occupational or speech therapy. The most common outcomes were feasibility and satisfaction, but tools used to measure them were inconsistent. Although 57% of studies demonstrated effectiveness of hybrid allied health services, none measured cost-effectiveness.

**Discussion:**

Despite the potential of hybrid allied health services for children and young people, the literature remains at a preliminary stage. Standardization of definitions and outcome measures, and clearer reporting of service characteristics and results would likely promote consolidation of hybrid allied health services in children and young people into clinical practice.

**Supplementary Information:**

The online version contains supplementary material available at10.1186/s12984-024-01401-1.

## Introduction

Over the past two decades, emerging developments in online technology offer a range of opportunities in support of remotely delivered healthcare (telehealth), including rehabilitation services provided by physicians and allied health professionals [1]. Telehealth is defined by the US Department of Health and Human Services as “the use of electronic information and telecommunication technologies to support long-distance clinical health care, patient and professional health-related education, public health and health administration” [2]. Telehealth can be provided synchronously (i.e., when the therapist and patient interact remotely in real time) or asynchronously (i.e., when therapeutic content is stored and shared between therapist and patient not concurrently) [3].

Evidence from studies of adult patients suggests that allied health services, delivered remotely, are as effective as in-person service therapy [4, 5]. Similar reports exist for remote allied health interventions for children and young people with diagnoses such as autism, cerebral palsy (CP), acquired brain injury (ABI), and developmental delay [1, 6, 7] as well as those with multiple disabilities [8]. Remote health service delivery provides improved access for people who are geographically distant from healthcare [9]. Remote health service delivery also provides unique opportunities for interaction with different family members and understanding the familial context of the child, thus enhancing engagement of families in treatment [10] and promoting the delivery of family-centered services [1, 11]. However, remote service delivery for children with disabilities carries with it many challenges. For example, remote care requires more attention from the caregiver who is physically with the child/adolescent – particularly in terms of physical touch and technical assistance with equipment. Furthermore, remote service delivery is challenging since it involves an increased need to engender the child’s motivation and cooperation, particularly for younger children [6]. Due to the complexity of the rehabilitation context for children and young people, the investigation of remote allied health services (allied health services provided where allied health professional and child or young person are not in the same physical setting) [1] in this group is especially important [12].

The unique challenges described here likely underlie the limited uptake of remote allied health services until the outbreak of the COVID-19 pandemic [13], a “tipping point” [14] in the adoption of telehealth worldwide. During COVID-19, many medical centers deployed remote assessments and interventions in various allied health domains (physical, occupational and speech and language therapy) in order to maintain continuity of high-quality care to children and families [15–17]. Nevertheless, the many difficulties associated with remote allied health services (e.g., technology, physical distance, environmental distractions) [12] are responsible for the rapid return to in-person rehabilitation service delivery after the pandemic subsided [18].

A potential solution for achieving fuller integration of remote services may be the use of hybrid allied health services, which are services provided by allied health professionals, in a combined manner: both in person as well as remotely. In order to overcome some of the challenges associated with remote care, hybrid allied health services are emerging as a promising approach for both adults, children and young persons [19]. Hybrid allied health services take advantage of the benefits of enhanced access to treatment in a child’s natural environment (such as goals appropriate for home environment, use of home accessories for treatment ). Hybrid allied health services may even become the norm in the future [6]. However, the first step in understanding the potential for hybrid allied health services is to map existing evidence and establish a framework for describing hybrid allied health services for children and young people. In this work, children and young people are defined as aged 0–21, given that this is an age where young people in special education are still expected to receive a relatively high dose of allied health services. A scoping review methodology was chosen given the breadth of the evidence and the multiple knowledge gaps in the field [20].

The objectives of this study were to document 1. the definitions, 2. service characteristics, 3. outcome measures and 4. results obtained using hybrid allied health services for children and young people. This study is part of a larger research project, registered in the Open Science Framework repository (https://osf.io/hr4vx/), designed to examine existing definitions, models, outcome measures and results of hybrid allied health services for people across the life span. Based on the importance of mapping existing evidence to establish a framework for describing hybrid allied health services for children and young people, the research question was, “What are the definitions, service characteristics, outcome measures, and results of hybrid allied health services for children and young people?”

## Methods

A scoping review methodology was used following Arksey and O’Malley’s [21] five main stages: 1. identifying the research question – based on the rationale presented and the need to establish a framework for describing hybrid allied health services for children and young people, 2. identifying relevant studies, 3. study selection, 4. charting the data, and 5. collating, summarizing and reporting results. We refined the methodology as recommended by Levac et al. [22]. For example, the team met regularly during the study selection phase to discuss criteria for inclusion and exclusion as an iterative process. Consensus for study selection was reached after two independent team members selected the papers for inclusion; the data charting form was collectively developed by all team members and refined during the study selection process.

A rigorous and iterative search was carried out in February 2022 in multiple databases: Medline (Ovid), Embase, CINHAL, Psycinfo, Cochrane CENTRAL and Web of Science. After consultation with a librarian, the key words included various terms for allied health services (e.g., rehabilitation, relevant therapies, psychology, and social work) together with the word “hybrid” or “blended”. In addition, we searched papers for additional keywords which were relevant. The detailed search strategy for the different databases is attached in Appendix 1.

Inclusion criteria (for the full study) were: (1) English language; (2) published in peer-reviewed journals and conference proceedings; (3) articles published since 2011; (4) participants were children or young people receiving healthcare services by allied health professionals; and (5) articles document a hybrid allied health service intervention. Exclusion criteria were: (1) articles in which interventions do not include professional monitoring of the remote rehabilitation component(s) (e.g. an exercise program that is given for at-home training, but its performance is not documented); (2) systematic reviews and meta-analyses; and (3) medical, nursing or educational interventions. Covidence software (Covidence, 2018) was used to perform study selection and data extraction. Given that the current results are part of a larger research project, the studies included in this paper were those targeting children and young people, with a maximum age of 21 years since special education in many countries is provided up until this age. This selection was performed after the initial search ended.

Two experienced research assistants were trained to implement a two-stage selection process that consisted of screening of all publication titles and abstracts and then reviewing all relevant full-text articles. Two researchers (NG and TK) resolved with consensus issues related to article inclusion criteria during these stages. Following full-text review, the same research assistants separately extracted data according to a standardised form developed by the research team (NG, TK, PLW, LG and RK). Details on extracted data can be found in Tables 1 and 2. Conflicts were resolved by NG and TK together, following a single consultation. As recommended by Levac et al. [22], charted data were collated and summarized by the research team in an iterative extraction process. All data analysis procedures followed the PRISMA-ScR checklist [23].

**Table 1:** Study and service characteristics

Study ID	Country	No. participants	Age	Gender	Pathology	Study design	Profession	Treatment type	Hardware	Software	Synchronous/asynchronous	Who gets the treatment
Rasing et al. [37]	Netherlands	129	13–22, 16.6 (2.03)	106 F, 23 M	Depression	Quasi-experimental controlled	Psychology	CBT		Doepressie blended online CBT program	Both	Child
Gibbs et al. [24]	United States	4	5–12	4 M	Autism spectrum disorder	Case series	Occupational therapy	Sensory integration	Webcams	Website allowing internet conferencing	Synchronous	Parent/caregiver
Tripicchio et al. [25]	United States	64	9.6 (3.1)	21 F, 43 M	Obesity	Non-randomised experimental study	Dietary	Family based behavioral group	Tablets (Apple Ipad)	FITNET a physical activity app, Skype	Both	Both
Hilyard et al. [34]	Australia	11	Hybrid: 14.4–17.8, Control: 12.1–16.6	10 F, 1 M	Chronic pain	Randomised controlled trial	Physical therapy; Occupational therapy; Psychology	Interdisciplinary- motor and CBT		SCOPIA videoconferencing	Synchronous	Both
Benz et al. [35]	Australia	54	13 [10–15]	35 F, 19 M	Cystic fibrosis	Retrospective cohort study	Physical therapy	Respiratory	Smart phone, tablet or laptop		Synchronous	Child
Rauschenberg et al. [38]	Netherlands	10	14–25, 20.3 (3.8)	7 F / 3 M	Psychosis/ Depression/ Anxiety	Uncontrolled pilot study	Psychology	CFI- compassion focused intervention (third-wave CBT)	Smartphone	mHealth app-EMIcompass (psyMate, Psymate BV), email and phone	Asynchronous	Child
Hollmann et al. [42]	Germany	9	7–17, 14.11 (3.29)	2 F, 7 M	Obsessive compulsive disorder	Single armed feasibility design	Psychotherapy	CBT	Computer with webcam and speakers, smartphone	teleconferencing system video, smartphone app, online cloud (BW sync &Share)	Both	Both
Sankar et al. [26]	United States	13	17–24, 20.5 (1.9)	10 F, 3 M	Bipolar disorder	Randomized experimental study	Psychotherapy	Interpersonal and social rythm therapy (IPSRT)		secure video platform- BE-SMART-DR	Synchronous	Child
Fleischman et al. [27]	United States	40	10–17, 14.4 (1.9)	31 F, 9 M	childhood Obesity	Randomised controlled trial	Psychology; Dietary	nutrition education and- dietary and physical activity counseling. Psychologist- cognitive behavioural	Webcams, tablets (IPads with 3G Internet)	Video Desktop	Synchronous	Both
Hooshmand et al. [28]	United States	222	9.8 (5.1)		Children with special needs	prospective quasi-experimental	Dietary; neurologist, nurse, registered practitioner,	neurology, and nutrition		Polycom	Synchronous	Parent/caregiver
Stasinaki et al. [43]	Switzerland	31	10–16, mean 13.6	13 F, 18 M	Obesity	Randomised controlled trial	Dietary; Counseling	multicomponent BCI (behavioral change intervention)	Smartphone	pathmate2 app- nutritional education and physical activity	Both	Child
Fonseca et al. [44]	Portugal	80	12–18 ,14.6 (1.88)	41 F, 39 M	Overweight	Randomised controlled trial	Psychology; Dietary; exercise physiologist and pediatrician	weight management- exercise and educational		NextStep – online platform (resources, self-monitoring, social support, interactive training and motivational tools	Both	Child
Sehlin et al. [40]	Sweden	44	Hybrid: 15–32, 21 (5.1) Control: 15–32, 22.1 (5.1)	21 F, 23 M	Neurodevel- opmental disorders- ADHD and ASD	Non-randomised experimental study	Occupational therapy; Psychology; social workers, and special education teachers	Support regarding aspects of daily life	computer	chat program	Synchronous	Child
Rasing et al. [36]	Netherlands	129	13–22, 16.6 (2.03)	106 F, 23 M	Depression	Quasi-experimental controlled	Psychology	CBT		Doepressie blended online CBT program	Both	Both
Armbrust et al. [39]	Netherlands	64	8–13, 10 (1.4)	41 F, 23 M	Juvenile idiopathic arthritis	Randomised controlled trial	Psychotherapy	educational and cognitive behavioral program	Computer	Rheumates@Work – an interactive, educational, and cognitive behavioral program	Both	Both
Shahnavaz et al. [41]	Sweden	18	8–15, 11 (2)	11 F, 7 M	Dental anxiety	Uncontrolled experimental study	Psychology	CBT		Online platform for internet based psychological treatments	Both	Both
VanderStoep et al. [29]	United States	223	5.5–12.9	163 M, 60 F	Attention Deficit hyperactivity disorder	Randomised controlled trial	Psychiatrists	caregiver behavior training and pharmacotherapy	computer	videoconferencing, web portal	Both	Parent/caregiver
Silk et al. [30]	United States	34	9–14, 11.4 (1.62)	17 M, 17 F	anxiety	Uncontrolled experimental study	Psychology; licensed professional counselor	CBT	smartphone	SmartCAT - an mHealth platform	Asynchronous	Child
Ryer et al. [31]	United States	1	8	1 M	Stuttering	Case report	Speech language pathology	Smooth Speech Treatment CBT	Computers, speakers/ headphones with microphones and webcams	videoconferencing - adobe connect, switched to zoom for last 5 sessions	Synchronous	Child
Timpe et al. [32]	United States	3	3.7, 4.5, 5.6	1 F, 2 M	Down’s syndrome (with severe speech impairment)	Case series	Speech language pathology	Augmentative and alternative communication (AAC) partner instruction program	mobile AAC technology (apple ipads), mobile phones	TouchChat HD with WordPowerVR 2 communication application	Both	Parent/caregiver
Myers et al. [33]	United States	223	5.5–12.9	163 M, 60 F	Attention Deficit hyperactivity disorder	Randomised controlled trial	Psychiatrists	caregiver behavior training and pharmacotherapy	computer	videoconferencing, web portal	Both	Both

**Table 2:** Outcome measures used in the included studies

Area	Construct	Measure	No. of studies
Program Evaluation	Program feasibility assessment	Program completion [24, 39], participation in online sessions [24, 25], using learned strategies [32], recruitment [38], retention [26], compliance [38], acceptability [25, 38], attendance [25, 26], usage [25], System Usability Scale (SUS) [30], Client Evaluation of Services Questionnaire (CSQ-8) [30], no. of technical problems [39]	7
	Client satisfaction	Client Satisfaction Questionnaires [26, 30, 38, 39, 42], overall feelings [24] questionnaire for the evaluation of the treatment (FBB) [42]	6
	Therapeutic participant-therapist alliance and interaction	Working Alliance Inventory (WAI) [26], e-mails and chat sessions with instructors [39]	2
	Professionals’ feedback	Caring Professional Scale (CPS) [32], The Summary Therapist Feedback Form (STFF) [42]	2
	Cost	Costs for clients: The Family Cost Survey (Researcher developed, Nonstandard) [32], monitoring the financial consequences for the participants [39], distance from home to hospital [34, 35], and travel related expenses (time, missed work) [34]. Costs for service provider: cost of program development, staff costs [39].	4
	Safety	Documenting adverse events [34, 35, 38]	3
Parent/Family	Family centered care	The Measure of Process of Care-20 (MPOC-20) Item Scale [32]	1
	Parenting stress/strain	The Parenting Stress Index (PSI) [29], The Caregiver Strain Questionnaire (CSQ) [29]	1
	Family empowerment and social validation	The Family Empowerment Scale (FES) [29], Social validation- parents perspectives questionnaire [32]	1
Physical	Physiological data	Heart rate, electrodermal activity - app linked to a special wristband (empatica) [42], Blood pressure - auscultation [26, 43]	3
	muscle and fat mass	Bioelectrical impedance analysis [43], Waist circumference and triceps skinfold-Gulick measuring tape and Lange caliper [27, 43]	2
	BMI	Weight, height, BMI (kg/m2) [25, 27, 43, 44]	4
	Physical functioning	Functional Disability Index (FDI) [34], modified Dordel-Koch test plus plate tapping from eurofit test [43], interview [27]	3
	Sensory processing skills	The Sensory Processing Measure (SPM) [24]	1
	Dietary intake	Interview [27], Adherence to Weight Control Questionnaire (AWCQ) [44]	2
	Lung function	Spirometry, ppFEV [35]	2
	Pain intensity	Pain numerical rating scale [34] PROMIS Pediatric Pain Interference Scale (one question related to sleep) [34]	1
Affective	diagnostic and clinical assessments	Children’s Yale-Brown Obsessive Compulsive Scale (CY-BOC) [42], The Schedule for Affective Disorders and Schizophrenia for School-Age Children Present and Lifetime Version(K-SADS-PL) [42], Clinical Global Impressions-Severity (CGI-S) [42], The Children’s Global Assessment Scale (CGAS) [42], Child Obsessive-Compulsive Impact Scale(COIS-RC) [42], The Child Behavior Checklist (CBCL/18) [42], Screen for Child Anxiety Related Emotional Disorders (SCARED) [30, 42], Schedule for Affective Disorders and Schizophrenia in School-Age Children Present and Lifetime version (K-SADS-PL) [30], Kiddie-Schedule for Affective Disorders and Schizophrenia, Present and Lifetime version (KSADS-PL) [36, 37], The Prodromal Questionnaire (PQ) [38]	5
	Depression / Anxiety	Child Depression Inventory-2 (CDI-2) [36, 37], The Patient Health Questionnaire (PHQ-9) [29], Hamilton Depression Rating Scale and Young Mania Rating Scale [26], Brief Symptom Inventory (BSI) [38], Symptom Questionnaire-48 [38], Hospital Anxiety and Depression Scale (HADS) [40], The Montgomery-ֳ Åsberg Depression Rating, Scale-Self-reported (MADRS-S) [40], Revised Child Anxiety and Depression Scale [34]	7
	Internalizing and externalizing symptoms	Youth Self Report scale (YSR) [36, 37]	2
	Propensity for suicidal behavior	Self-report Questionnaire Suicide Risk Taxation (SRT) [36, 37], The Suicide Propensity Subscale of the Concise Health Risk Tracking (CHRT) [26]	3
	Regularity of social rhythms	Brief Social Rhythm Scale (BSRS) [26]	1
	Ability to cope with stress	The Sense of Coherence (SOC 29) [40]	1
	Avoidance behaviors	Parent and Child Picture-Guided Behavior Avoidance Test (PG-BAT) [41]	1
	Fear	Fear of Pain Questionnaire (FOPQ) [34], CFSS-DS child and parental versions [41]	2
General function	Skill acquisition measures	Global Assessment of Functioning scale [40], Penn Emotion Recognition Task [30], Ambiguous Situations Questionnaire (ASQ) [30], The Child Version of the Skill Acquisition Measure (SAM-C) [30] Columbia Impairment Scale, Parent-Report Version (CIS-P) [21]	2
	Quality of Life	Impact of Weight on Quality of Life-Lite (IWQOL-Lite Questionnaire) [44], Manchester Short Assessment for Quality of Life (MANSA) [44]	1
	Self-efficacy	Child Self Efficacy Scale (CSES) [34], Self-Efficacy Questionnaire for Specific Phobias (SEQ-SP) [41], The Rosenberg Self-Esteem Scale (RSES) [40]	3
	Positive life perspective and Health responsibility	Adolescent Lifestyle Profile(ALP-R) [44]	1
Measures for Specific Conditions	Stuttering	Stuttering Severity Instrument, Fourth Edition (SSI-4) [31], The Overall Assessment of the Speaker’s Experience of Stuttering (OASES) [31]	1
	Dental Phobia	The phobic disorders supplement included in The Kiddie Schedule for Affective Disorders and Schizophrenia (K-SADS-PL) [41], research developed [41]	1
	Child ADHD symptoms	Vanderbilt ADHD Diagnostic Parent Rating Scale (VADRS-Parent) [29, 33]	2
	Communication	Turn taking rates [32], frequency of novel semantic concepts [32]	1

## Results

### Characteristics of reviewed studies

**Fig. 1:**
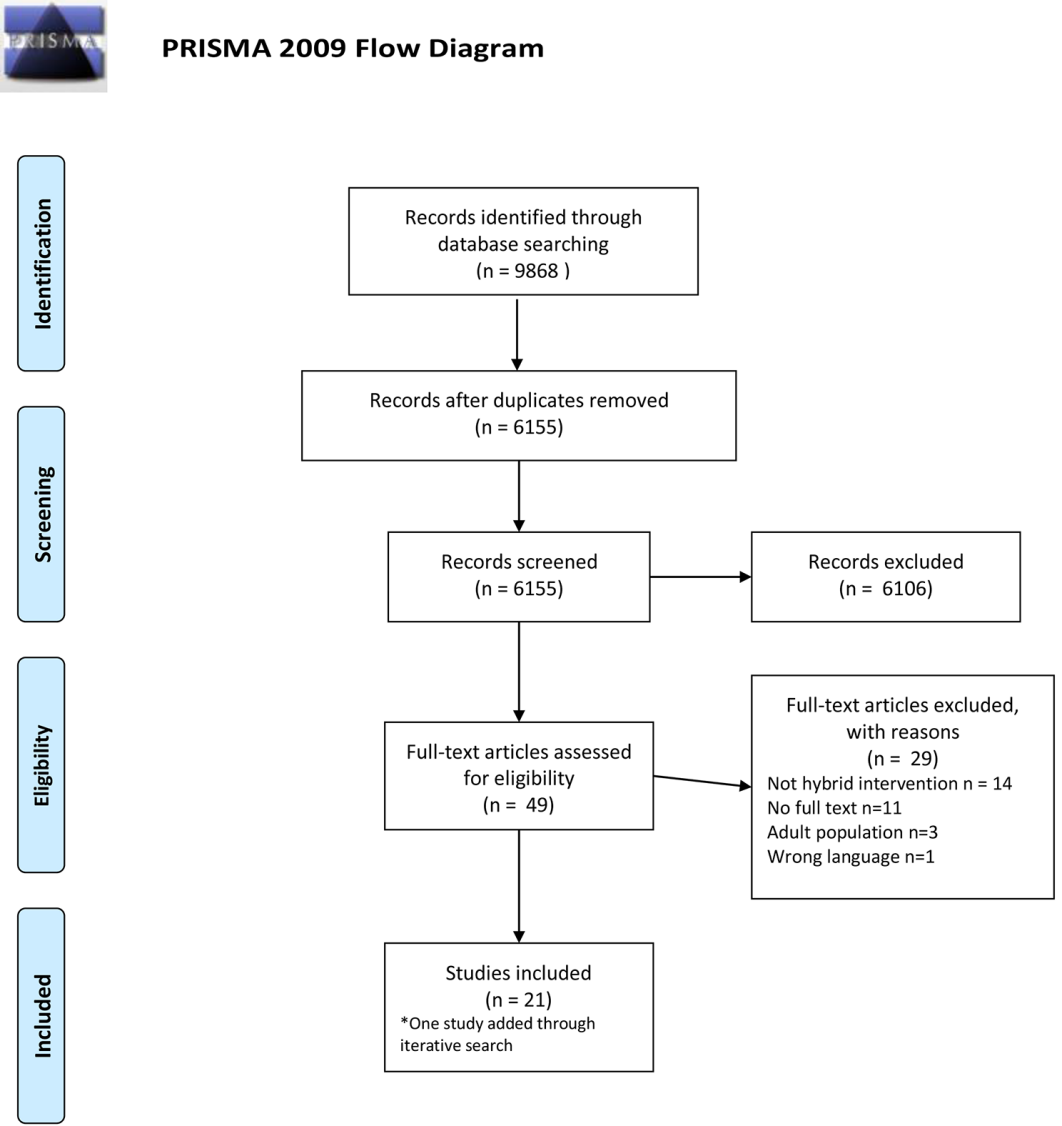
PRISMA diagram showing the number of articles reviewed throughout the selection process

**Fig. 2:**
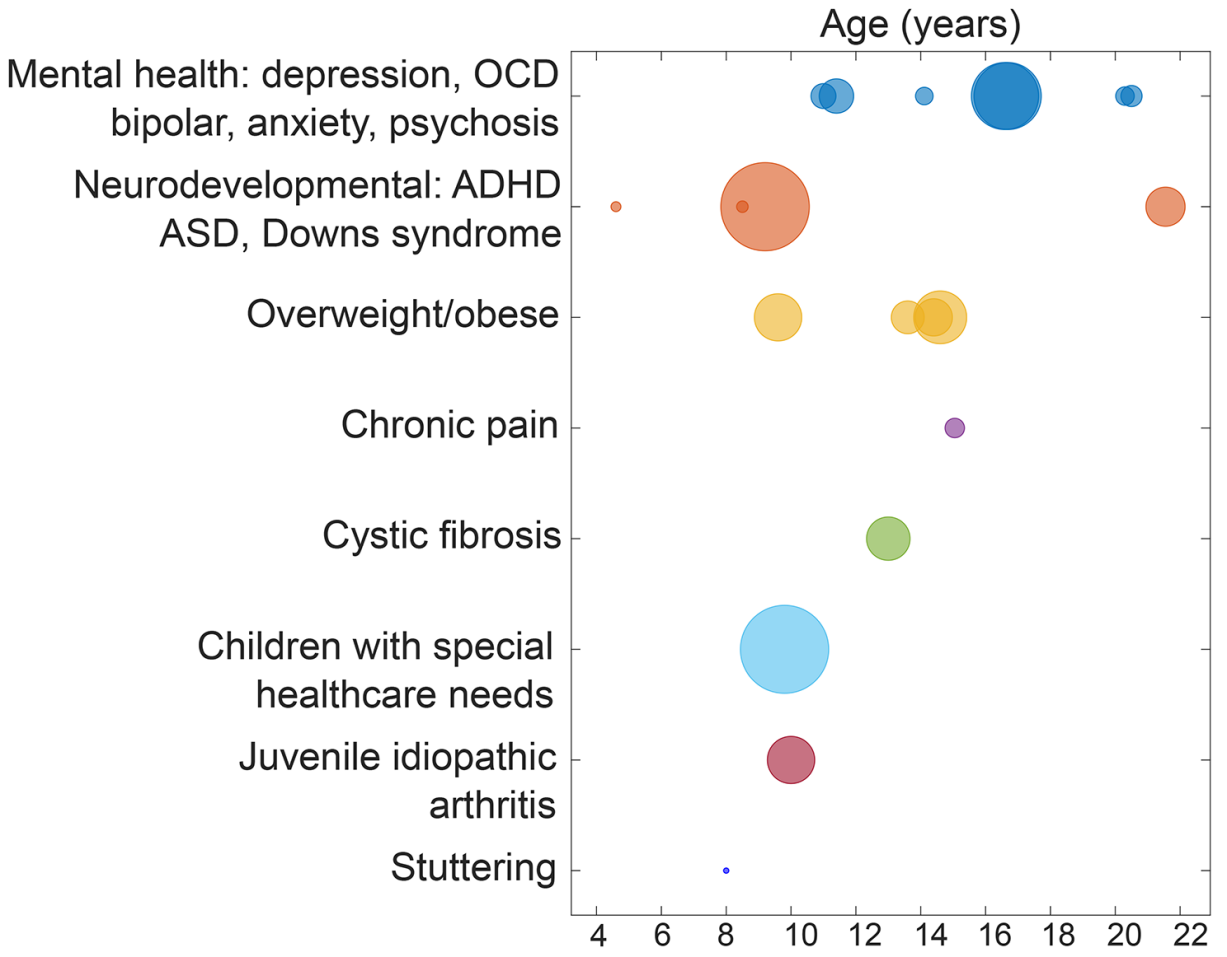
Bubble plot of ages and sample sizes for the studies according to the different categories of population. Each bubble represents a single study, where the bubble size represents the study’s total sample size

Out of the 9,868 identified studies, *n* = 21 were included in this scoping review. The selection and exclusion process are presented in the PRISMA chart (Fig. 1). Study characteristics are summarized in Table 1. Ten out of the 21 studies (*n* = 10, 48%) originated in the United States [24–33], two studies (*n* = 2, 10%) originated in Australia [34, 35], and the remaining nine studies (*n* = 9, 42%) originated in Europe including the Netherlands [36–39], Sweden [40, 41], Germany [42], Switzerland [43] and Portugal [44]. Study participants’ mean ages ranged 4–21 years. Sample sizes ranged from 1 to 223 participants (Fig. 2). The most common interventions were for mental health conditions (*n* = 7, 33%) [26, 30, 36–38, 41, 42], followed by neurodevelopmental conditions (*n* = 5, 24%) [24, 29, 32, 33, 40] and obesity/overweight (*n* = 4, 19%) [25, 27, 43, 44].

Most studies (*n* = 17, 81%) used experimental or quasi-experimental designs. A control group was included in 13 studies [25, 27–29, 33–37, 39, 40, 43, 44], over half of which (8 studies) used randomization for group assignment [26, 27, 29, 33, 34, 39, 43, 44]. Three papers were case reports or case series [24, 31, 32] and one study [35] was a retrospective observational study.

### Objective 1: definitions of hybrid allied health services in children and young people

Conceptual definitions of hybrid allied health services, when provided, included services delivered via a combination of traditional in-person treatments and service delivered via technologies such as telephones, video-conferencing and applications, and referred to as telehealth, eHealth or tele-practice [29, 31, 32, 34, 36, 37]. While most studies used the term “hybrid” healthcare, some used “blended” treatment [36, 37] or referred to “technology adjuncts” to treatment [25].

Operational definitions (e.g., ratio of remote/in-person), derived from deployment of services, also varied substantially. For studies where this information was available [24–28, 31, 32, 34, 35, 40, 42, 43], the ratio of remote to in-person treatment time was computed; the mean ± standard deviation ratio equaled 57.8% ± 25.0% (i.e., somewhat more time spent in remote therapy but with wide variations in the relative amounts). For most of the studies with a ratio of less than 50% (i.e., less remote therapy) the hybrid components involved both synchronous and asynchronous interactions (e.g [25, 43]) however, in general, data on the duration of the asynchronous component was not reported.

The remote content of the intervention was, in most cases (*n* = 16, 76%) [25–27, 29–33, 35–37, 39–41, 43, 44], intertwined with in-person treatments throughout the entire duration of the intervention, either as synchronous sessions or asynchronous activities. Eight (*n* = 8,38%) of the 21 studies used synchronous interaction in the remote part of the hybrid allied health service [24, 26–28, 31, 34, 35, 40], four (*n* = 4,19%) used asynchronous remote interactions [30, 36–38], and the rest [25, 29, 32, 33, 39, 41–44] combined both synchronous and asynchronous components. Activities delivered during the intervention varied according to the study aim, such as an asynchronous online Cognitive Behavioral Therapy (CBT) program for depression [36, 37], online visits with obesity specialists [27] or parent consultations with occupational therapists [24]. In five studies (*n* = 5, 24%) [24, 28, 34, 38, 42], the remote component was introduced in the middle or towards the end of the intervention. Importantly, in no study was the initial session remote.

### Objective 2: service characteristics

As presented in Table 1, the reviewed studies described therapies by diverse health professionals. Out of the 21 studies, 15 (*n* = 15, 71%) [26, 27, 29, 30, 33–42, 44] involved mental health professionals (psychologists/psychotherapists) as part of the team delivering the intervention. Psychological support was either a sole intervention (*n* = 4 studies, 19%) [36–38, 41] or part of a multidisciplinary intervention (*n* = 4 studies, 19%) [27, 34, 40, 44]. Additional studies involved psychotherapists [26, 39, 42], psychiatrists [29, 33] and licensed counsellors [30]. Within these 15 studies related to mental health professionals, 10 (*n* = 10, 71%) [27, 30, 34–39, 41, 42] used CBT as the main therapeutic tool. Although social work was included in our search, only one study (4.8%) involved social workers who provided the CBT intervention [40]. An additional study [31] used CBT, administered by a speech and language pathology graduate student. Dietary interventions were included in five studies (*n* = 5, 24%) [25, 27, 28, 43, 44], occupational therapy was provided in three studies (*n* = 3, 14%) [24, 34, 40] working with children with autism, ADHD and pain, physical therapy was provided in two studies (*n* = 2, 9.5%) [34, 35] working with children with pain and Cystic Fibrosis, and speech and language pathology was provided in two studies (*n* = 2, 9.5%) [31, 32] working with children with stuttering and down syndrome.

It is noteworthy that most hybrid allied health services identified in this review targeted psychological wellbeing goals, i.e., maintaining and nurturing one’s mental health. These interventions may be particularly suited to the hybrid framework given their dependence on verbal interactions. A dominant intervention approach for the reviewed hybrid studies was CBT, demonstrating the importance of this approach as an effective psychological intervention [45] in different populations. In contrast, the prevalence of physical, occupational and speech therapy within the identified studies of hybrid allied health services was surprisingly low.

The studies identified in this review also present a wide range of intervention durations. Intervention duration was longer than 15 weeks in 5 studies [27, 29, 31, 33, 43], less than 5 weeks in 3 studies (*n* = 3, 14%) [24, 32, 35], and from 5 to 15 weeks in the remaining studies [25, 28, 30, 34, 36–41, 44] .

The studies included in this review showcased a broad spectrum in software and hardware diversity. Customized software, exclusive and not commercially accessible, was utilized in *n* = 16 studies (76%) [24, 26–30, 33–41, 43]. In contrast, hardware diversity was less prominent, predominantly relying on commercially available options: computers were employed in *n* = 16 studies (76%) [24, 26, 28–31, 33–37, 39–42, 44], smartphones in *n* = 12 (57%) [26, 32, 34–39, 41–44], and tablets in ten (*n* = 10, 48%) [25–27, 32, 34–37, 41, 44]. Seven studies (*n* = 7, 33%) [26, 34–37, 41, 44] integrated a combination of computers, tablets, and smartphones.

Accessibility of hybrid allied health services is an important factor in achieving scalability of at-home therapy to a large group of potential users [46]. To this end, the current results documented that the hardware consisted of, for the most part, “off the shelf” (smartphones, tablets, computers), devices that are often available to families. In contrast, in over 75% of the studies, the software was designed for a specific protocol, and scalability was not discussed in terms of the cost, technical support or training for use by other clinical settings. The extent of training to learn how to operate the software or hardware varied. When provided, it included topics such as how to set up and run the software correctly, position the camera and microphone, or don any wearable device to ensure clear communication during the remote sessions.

### Objective 3: outcome measures

This scoping review identified two main types of outcomes: (i) clinical change assessment and (ii) program evaluation including client satisfaction. All outcome measures are described in Table 2. Outcomes assessing clinical change were condition-specific and addressed the perspectives of children and young people, and their parents and/or family members. Child outcomes included physical [24–27, 34, 35, 42–44] and affective [26, 29, 30, 34, 36–38, 40–42] components, general functional measures related to skill acquisition [30, 40], quality of life [44], self-efficacy [34, 40, 41], life perspective [44], and measures related to specific conditions [29, 31–33, 41]. Parent and family outcomes [28, 29, 32] included family-centred care, parental stress or strain, family empowerment, and social validation. The diversity of outcome measures demonstrates the wide range of health needs or conditions that hybrid allied health services can address.

With regard to non-clinical measures, the most commonly-reported outcomes were client satisfaction [24, 26, 30, 38, 39, 42] and feasibility and acceptability [24–26, 30, 32, 38, 39].

One non-clinical outcome which is often considered as important is cost-effectiveness. However, this scoping review showed that it was rarely measured. Only four papers (*n* = 4,19%) reported on factors associated with cost [28, 34, 35, 39], and none measured cost-effectiveness. Within the four studies, cost was measured differently. Armbrust et al. [39] calculated the cost of program development, salaries for the treating staff and family expenses such as travel and babysitter support. Hilyard et al. [34] estimated time and cost of travel to and from a hospital over a two-month period, the number of hours spent at the clinic for each trip as well as missed employment days by family members. Benz et al. [35] measured travel time and distance saved by using remote intervention as compared to travel of the therapist to the patients’ homes. Hooshmand et al. [28] created the Family Cost Survey, a customized survey of cost including items related to travel, transportation, loss of wages, child care, food, and lodging costs related to the family’s visit to the clinical site. The survey also included questions regarding the anticipated cost to the parent/guardian for clinical visits with specialists if telehealth was not available.

### Objective 4: results of studies evaluating hybrid allied health services

Among the 21 studies reviewed, only *n* = 13 (62%) incorporated a control group. These control groups encompassed in-person treatments [25, 27, 28, 34–37, 43], and/or treatment as usual [36, 37, 40, 44] or a lower dose (one session) of remote within the hybrid allied health services [29, 33].

Notably, four studies (*n* = 4, 19%) [27, 29, 33, 40] showcased significant between-group differences favoring hybrid allied health services for at least one outcome measure. Conversely, a single study [43] indicated significant short-time between-group differences in favor of the control group—an in-person counseling intervention—for one specific outcome among several assessed. The tele-consultation was provided via a virtual agent and not a person as was the case in the other studies, and therefore may have been less personalized to the participants’ needs. Nevertheless, in this study, both groups improved in other outcome measures indicating that the hybrid allied health service was not inferior to conventional treatment. Within a project presented in two papers, multiple treatments of remote allied health professionals, in combination with in-person treatments, demonstrated significant enhancements for both child outcomes [33] and parental outcomes [29].

Significant within-group (pre-post) differences following hybrid allied health services were reported in three studies (*n* = 3, 14%) [25, 43, 44] and one study (*n* = 1, 4.8%) [35] reported an improvement without statistical testing. Among the eight non-controlled studies, five (*n* = 5, 63%) [26, 30, 38, 41, 42] showed statistically significant improvements. Three studies (*n* = 3, 37%) [24, 31, 32] reported improvements using descriptive statistics only (not inferential). Furthermore, reported satisfaction with hybrid allied health services was consistently rated as good-to-excellent [25–28, 30–32, 34, 39, 41, 42] as were acceptance and retention (adherence) rates [24, 26, 38, 42, 43].

## Discussion

Hybrid allied health services models may be a feasible and potentially effective way to alleviate many of the accessibility and financial challenges in healthcare [19]. However, this scoping review has highlighted several key limitations of hybrid allied health services research in children and young people, including a lack of consensus regarding definitions of hybrid allied health services and implementation of service models. In addition, there is a relatively high prevalence of studies based on conversational/verbal interventions (i.e. psychology) versus the infrequency of other types of “hands-on” remote treatments (e.g., physical, occupational and speech therapy). This may be due to the conversational nature of verbal interventions that limit the clinicians’ exposure to challenges associated with remote therapy and assessment. Results for hybrid allied health services were generally favorable, despite the relatively low methodological rigor (e.g., the lack of a control group in *n* = 8, (38%) of the studies), and the large number of inconsistently defined outcome measures across the different studies.

The current work identified widespread diversity in both conceptual and operational definitions associated with hybrid allied health services in children and young people. This made it difficult to consolidate the scoping review’s search strategy efficiently. The inconsistency of terms as well as the variety of operational definitions confounded comparisons, for example, with respect to frequency and duration of in-person versus remote treatment in the hybrid allied health services – especially in studies where the intervention was asynchronous. Within the variability of program characteristics, a consistent finding was that by cconducting the initial session(s) in-person (i.e., not remotely) the therapeutic alliance was facilitated [47], entailing agreement between therapist and patient about the goals of therapy and the steps needed to achieve these goals. It also supported a patient-therapist bond that improved coping with challenges as they arose during the therapeutic process [48, 49]. These accomplishments are then nurtured remotely. Indeed, the literature acknowledges that therapeutic alliances can be maintained remotely [10, 47], specifically during extreme conditions such as the pandemic [15, 16]. Additionally, initial in-person sessions allow for instruction of equipment usage and serve as means of troubleshooting technological issues early on [27]. Moreover, in-person sessions allow clinicians to perform physical assessments that are often more challenging to perform remotely [10]. Thus, it is recommended that guidelines for planning hybrid allied health services should include an initial period of in-person meetings (one or more) to accommodate these factors.

Hybrid allied health services described in the current study varied in care delivery as well as in the population targeted. Some treatments, such as those which require monitoring motor and cognitive performance and hands-on manipulation during treatment, may pose a greater challenge for remote services [50]. In younger children, such activities are even more challenging since the active participation of caregivers is crucial for enabling most remote treatments, whereas in-person treatments may or may not include a caregiver. Interestingly, allied health professionals reported that engaging children and young people remotely is more challenging then in-person, but engaging their parents becomes easier when remote [51].

Another source of variability was identified in the duration of care delivery which may be related to the specific needs and goals of the population or to the technology used. For example, intensive synchronous treatments involving therapists may be more difficult to implement for a longer period of time compared to the use of automated conversational agents.

With respect to technology usage, the current results demonstrated that while most studies relied on “off-the-shelf” hardware, the software used was, in most of the cases, customized for the specific study, and training required to use the software was varied. Provision of clear and concise instructions and guidance to patients and family members when introducing new software or digital tools is an essential requirement for successful home-based treatment. It reduces the likelihood that technical barriers will prevent patients from using the software effectively to derive the most benefit from treatment. For example, the Department of Physical Medicine and Rehabilitation at Johns Hopkins Hospital established protocols for providing training in the use of basic technologies (phone, Internet connectivity, telemedicine literacy and, more rarely, virtual gaming platforms) [52]. Based on their experience during COVID-19, use of standardized equipment and “off the shelf” software entailed a level of training and accommodation that is feasible for most patients. Such protocols appear to reduce dissatisfaction with telehealth services and can increase the scalability of hybrid allied health services solutions across the continuum of care. In recent years, efforts are being made, via collaboration between clinicians, families and healthcare systems, to support innovation in service implementation in order to maintain a high level of engagement with remote allied health services [53].

Documenting the effect of hybrid allied health services is complex, and the current study demonstrated that the outcome measures used in the different studies vary substantially. The acceptability of hybrid allied health services can be evaluated using Sekhon et al.‘s [54] Theoretical Framework of Acceptability (TFA), which defines acceptability as “a multi-faceted construct that reflects the extent to which people delivering or receiving a healthcare intervention consider it to be appropriate, based on anticipated or experiential cognitive and emotional responses to the intervention” (page 4). Dostie et al. [55] determined that the term “acceptability” was inconsistently defined in different studies, and recommended selection of overarching terms to reach consensus on outcome measure terminology for future program evaluations. The wide range of outcome measures used in studies here supports this recommendation. This does not refer only to acceptability, as additional program evaluation outcomes included achievement of a therapeutic alliance [26, 39], feedback from professionals on the service [28, 42], cost [28, 34, 35, 39] and safety [34, 35, 38]. Most of these outcome measures were non-standardized, having been developed for a particular study. More consistent use of program evaluation measures in future studies will help to compare programs, draw conclusions regarding effectiveness and, eventually, develop consensus guidelines for future hybrid allied health services.

## Limitations

The current study has several limitations. First, we limited our search to studies in the English language hence missing possibly relevant literature published in other languages. As the results presented in this paper were part of a larger study assessing hybrid health services across the life span, no search terms specifically related to children were included at the initial stage. Furthermore, as this study was a scoping review, it was not geared to assess the quality of the studies evaluated. Future reviews that include studies with clearly defined, cohesive objectives and outcomes, will further advance the literature on hybrid allied health services models.

### Recommendations for future research

Our findings support the need to further research pediatric hybrid allied health services models that will address the following recommendations: First, additional research is needed in the fields of physical, occupational and speech therapy, where remote interventions have often been shown to be effective and to maintain a therapeutic alliance [15, 16]. Additional research should focus on evaluating effectiveness across diverse conditions and practice settings, assessing service accessibility and equity, exploring innovative technologies, addressing provider training, and investigating cost-effectiveness and impact on regulatory practices and policy considerations. Second, our findings highlight the advantages of considering initial in-person sessions when designing hybrid allied health services. This approach facilitates a seamless transition to the technological environment while fostering a stronger therapeutic alliance. Moreover, the planning and reporting should address the ratio of in-person to remote sessions as well as duration of each format and type of remote intervention (e.g., synchronous/asynchronous). These will enable us to compare between studies, understand the association between service characteristics and effectiveness of intervention and to better translate knowledge into clinical practice. Furthermore, in the selection of a technological platform, prioritizing the accessibility and scalability of software and hardware becomes crucial. This involves reducing dependence on customized software or, if used, planning for its wider distribution after the study concludes. Given the continuously evolving technology landscape, each service setting should carefully create a foundational toolkit. This toolkit should encompass minimum software and hardware elements tailored to suit the needs of service recipients, providers, and the objectives of the hybrid allied health services they aim to deliver. While acknowledging the feasibility of hybrid allied health services and their satisfactory user acceptance, it is apparent that the incorporation of control groups and randomized assignment of participants to interventions has been limited. Looking ahead, there is a critical need to conduct more extensive randomized controlled studies. These studies should evaluate a spectrum of condition-specific outcomes alongside effectiveness outcome measures. This approach would significantly enhance our understanding of the true impact and efficacy of hybrid allied health services. Finally, outcome measures need to be standardized across studies, both when addressing specific clinical conditions as well as when evaluating acceptance, feasibility and cost-effectiveness. Specifically, while cost-effectiveness is often raised as an important rationale for remote treatments, its measurement is largely overlooked.

## Conclusions

Although hybrid allied health services are likely to become the norm in the near future [6], the literature on such interventions for children and young people remains at a preliminary stage. The literature to date tends to focus more on initial proofs of concept and less on the demonstration of program effectiveness. This review identified knowledge gaps in the field which, when addressed, should form the basis of establishing hybrid allied health services that incorporate the benefits of both in-person and remote interventions, maximizing cost-effectiveness and satisfaction of all stakeholders.

### Electronic supplementary material

Below is the link to the electronic supplementary material.

Supplementary Material 1

## Data Availability

The datasets analyzed during the current study are available from the corresponding author on reasonable request.
