# Correction: Hybrid approaches to allied health services for children and young people: a scoping review

**DOI:** 10.1186/s12984-024-01434-6

**Published:** 2024-08-10

**Authors:** Tal Krasovsky, Patrice L. Weiss, Liat Gafni-Lachter, Rachel Kizony, Naomi Gefen

**Affiliations:** 1 Department of Physical Therapy, Faculty of Social Welfare and Health Sciences, University of Haifa, 199 Abba Hushi Avenue, 3498838 Haifa, Israel; 2 Department of Pediatric Rehabilitation, The Edmond & Lily Safra Children’s Hospital, Sheba Medical Center, Ramat Gan, Israel; 3 Department of Occupational Therapy, Faculty of Social Welfare and Health Sciences, University of Haifa, Haifa, Israel; 4 The Helmsley Pediatric & Adolescent Rehabilitation Research Center, ALYN Hospital, Jerusalem, Israel; 5 College of Health and Rehabilitation Sciences, Department of Occupational Therapy, Sargent College, Boston University, Boston, USA; 6 Department of Occupational Therapy, Sheba Medical Center, Ramat Gan, Israel; 7 School of Occupational Therapy, Hebrew University, Jerusalem, Israel; 8 ALYN Hospital, Jerusalem, Israel

**Correction: Journal of NeuroEngineering and Rehabilitation (2024) 21:122** 10.1186/s12984-024-01401-1

Following publication of the original article [1], the affiliation details for Authors Liat Gafni-Lachter and Rachel Kizony were incorrectly given as ‘Liat Gafni-Lachter^4,5^, Rachel Kizony^4,6^’ but should have been ‘Liat Gafni-Lachter^3,5^, Rachel Kizony^3,6^’.

The original article has been corrected.
