# Supplementary material for: Hybrid approaches to allied health services for children and young people: a scoping review
Source: J Neuroeng Rehabil. 2024 Jul 19;21:122. doi: 10.1186/s12984-024-01401-1 (PMC11264746; doi:10.1186/s12984-024-01401-1)
Supplement: Supplementary file 1 — Supplementary Material 1 [file 12984_2024_1401_MOESM1_ESM.docx]

Appendix 1: Search strategy

**MEDLINE (Ovid)**

Database: Ovid MEDLINE(R) <1946 to February Week 1 2022>, Ovid MEDLINE(R) In-Process & In-Data-Review Citations <1946 to February 09, 2022>, Ovid MEDLINE(R) Epub Ahead of Print <February 09, 2022>

Search Strategy:

--------------------------------------------------------------------------------

1 ('allied health' or 'paramedical personnel' or 'rehabilitation' or 'neurorehabilitation' or telerehabilitation or 'tele-rehabilitation' or 'e-rehabilitation' or 'remote rehabilitation' or 'virtual rehabilitation' or 'functional assessment' or 'occupational therap*' or 'physiotherap*' or kinesiotherapy or exercise or 'muscle training' or 'speech language patholog*' or 'speech therap*' or 'language therap*' or 'music therap*' or 'art therap*' or dietitian* or dietetics or 'diet therapy' or 'dietary intervention' or audiolog* or hearing or psychologist* or therapist* or psychotherap* or telepsychology or 'tele-psychology' or 'social work*' or 'family therap*' or 'cognitive behavioral therapy').ti,hw,kf. or psychology/ or psychology, child/ or psychology, clinical/ or psychology, social/ or neuropsychology/

2 (hybrid or blended).ti,kf.

3 1 and 2

4 (telehealth or telemedicine or 'e-health' or ehealth or 'm-health' or mhealth or 'mobile health' or teletherapy or 'e-therapy' or telediagnosis or telepsychology or 'tele-psychology' or telerehabilitation or 'tele-rehabilitation' or 'e-rehabilitation' or 'remote rehabilitation' or 'virtual rehabilitation' or 'home-based' or ((web or online or internet or digital) adj2 (intervention* or treatment* or therapy or based)) or (mobile adj2 (app or application)) or videoconferenc* or 'video conferenc*').ti,hw,kf.

5 (combin* or integrat* or 'in conjuction' or hybrid or blend* or addition* or adjunct* or complement*).ti,ab,kf.

6 ('usual care' or 'care as usual' or standard or conventional or traditional or 'in-person' or 'face to face' or 'in-hospital' or 'hospital-based' or inpatient or 'in-patient' or 'in-clinic' or 'clinic-based' or 'center based' or 'centre based' or 'in-center' or 'in-centre' or onsite or 'on-site' or outpatient or 'out-patient').ti,ab,kf.

7 1 and 4 and 5 and 6

8 3 or 7

9 (protocol or qualitative).ti. or qualitative research/

10 8 not 9

***************************

**EMBASE**

Embase Search Strategy

| #1 | 'allied health':ti,de,kw OR 'paramedical personnel':ti,de,kw OR 'rehabilitation':ti,de,kw  OR 'neurorehabilitation':ti,de,kw OR telerehabilitation:ti,de,kw OR 'tele-rehabilitation':ti,de,kw OR 'e-rehabilitation':ti,de,kw OR 'remote rehabilitation':ti,de,kw OR 'virtual rehabilitation':ti,de,kw OR 'functional assessment':ti,de,kw OR 'occupational therap*':ti,de,kw OR 'physiotherap*':ti,de,kw OR kinesiotherapy:ti,de,kw OR exercise:ti,de,kw OR 'muscle training':ti,de,kw OR 'speech language patholog*':ti,de,kw OR 'speech therap*':ti,de,kw OR 'language therap*':ti,de,kw OR 'music therap*':ti,de,kw OR 'art therap*':ti,de,kw OR dietitian*:ti,de,kw OR dietetics:ti,de,kw OR 'diet therapy':ti,de,kw OR 'dietary intervention':ti,de,kw OR audiolog*:ti,de,kw OR hearing:ti,de,kw OR 'psychologist*':ti,de,kw OR telepsychology:ti,de,kw OR 'tele-psychology':ti,de,kw OR therapist*:ti,de,kw OR psychotherap*:ti,de,kw OR 'psychology'/de OR 'clinical psychology'/de OR 'cognitive behavioral therapy':ti,de,kw OR 'neuropsychology'/de OR 'child psychology'/de OR 'social psychology'/de OR 'social work*':ti,de,kw OR 'family therap*':ti,de,kw |
| --- | --- |
| #2 | hybrid:ti,kw OR blended:ti,kw |
| #3 | #1 AND #2 |
| #4 | telehealth:ti,de,kw OR telemedicine:ti,de,kw OR 'e-health':ti,de,kw OR ehealth:ti,de,kw OR 'm-health':ti,de,kw OR mhealth:ti,de,kw OR 'mobile health':ti,de,kw OR teletherapy:ti,de,kw OR 'e-therapy':ti,de,kw OR telediagnosis:ti,de,kw OR telepsychology:ti,de,kw OR 'tele-psychology':ti,de,kw OR telerehabilitation:ti,de,kw OR 'tele-rehabilitation':ti,de,kw OR 'e-rehabilitation':ti,de,kw OR 'remote rehabilitation':ti,de,kw OR 'virtual rehabilitation':ti,de,kw OR 'home-based':ti,de,kw OR (((web OR online OR internet OR digital) NEXT/2 (intervention* OR treatment* OR therapy OR based)):ti,de,kw) OR ((mobile NEXT/2 (app OR application*)):ti,de,kw) OR smartphone*:ti,de,kw OR 'smart phone*':ti,de,kw OR videoconferenc*:ti,de,kw OR 'video conferenc*':ti,de,kw |
| #5 | combin*:ti,ab,kw OR integrat*:ti,ab,kw OR 'in conjuction':ti,ab,kw OR hybrid:ti,ab,kw OR blend*:ti,ab,kw OR addition*:ti,ab,kw OR adjunct*:ti,ab,kw OR complement*:ti,ab,kw |
| #6 | 'usual care':ti,ab,kw OR 'care as usual':ti,ab,kw OR standard:ti,ab,kw OR conventional:ti,ab,kw OR traditional:ti,ab,kw OR 'in-person':ti,ab,kw OR 'face to face':ti,ab,kw OR 'in-hospital':ti,ab,kw OR 'hospital-based':ti,ab,kw OR inpatient:ti,ab,kw OR 'in-patient':ti,ab,kw OR 'in-clinic':ti,ab,kw OR 'clinic-based' OR 'center based':ti,ab,kw OR 'centre based':ti,ab,kw OR 'in-center':ti,ab,kw OR 'in-centre':ti,ab,kw OR onsite:ti,ab,kw OR 'on-site':ti,ab,kw OR outpatient:ti,ab,kw OR 'out-patient':ti,ab,kw |
| #7 | #1 AND #4 AND #5 AND #6 |
| #8 | #3 OR #7 |
| #9 | protocol:ti OR 'qualitative research'/de OR qualitative:ti |
| #10 | #8 NOT #9 |

**CINAHL**

CINHAL Search Strategy

| S1 | (TI "allied health" OR TI "paramedical personnel" OR TI rehabilitation OR TI neurorehabilitation OR TI telerehabilitation OR TI tele-rehabilitation OR TI e-rehabilitation OR TI "remote rehabilitation" OR TI "virtual rehabilitation" OR TI "functional assessment" OR TI "occupational therap*" OR TI physiotherap* OR TI kinesiotherapy OR TI exercise OR TI "muscle training" OR TI "speech language patholog*" OR TI "speech therap*" OR TI "language therap*" OR TI "music therap*" OR TI "art therap*" OR TI dietitian* OR TI dietetics OR TI "diet therapy" OR TI "dietary intervention" OR TI audiolog* OR TI hearing OR TI psychologist* OR TI therapist* OR TI psychotherap* OR TI telepsychology OR TI tele-psychology OR TI "social work*" OR TI "family therap*" OR TI "cognitive behavioral therapy" OR MW "allied health" OR MW "paramedical personnel" OR MW rehabilitation OR MW neurorehabilitation OR MW telerehabilitation OR MW tele-rehabilitation OR MW e-rehabilitation OR MW "remote rehabilitation" OR MW "virtual rehabilitation" OR MW "functional assessment" OR MW "occupational therap*" OR MW physiotherap* OR MW kinesiotherapy OR MW exercise OR MW "muscle training" OR MW "speech language patholog*" OR MW "speech therap*" OR MW "language therap*" OR MW "music therap*" OR MW "art therap*" OR MW dietitian* OR MW dietetics OR MW "diet therapy" OR MW "dietary intervention" OR MW audiolog* OR MW hearing OR MW psychologist* OR MW therapist* OR MW psychotherap* OR MW telepsychology OR MW tele-psychology OR MW "social work*" OR MW "family therap*" OR MW "cognitive behavioral therapy" OR (MH "Psychology+") OR (MH "Neuropsychology")) |
| --- | --- |
| S2 | (TI hybrid OR TI blended) |
| S3 | (TI telehealth OR TI telemedicine OR TI e-health OR TI ehealth OR TI m-health OR TI mhealth OR TI "mobile health" OR TI teletherapy OR TI telediagnosis OR TI telepsychology OR TI tele-psychology OR TI telerehabilitation OR TI tele-rehabilitation OR TI e-rehabilitation OR TI "remote rehabilitation" OR TI "virtual rehabilitation" OR TI home-based OR ((TI web OR TI online OR TI internet OR TI digital) N2 (TI intervention OR TI treatment OR TI therapy OR TI based)) OR (TI mobile N2 (TI app OR TI application)) OR TI videoconferenc* OR TI "video conferenc*") OR (MW telehealth OR MW telemedicine OR MW e-health OR MW ehealth OR MW m-health OR MW mhealth OR MW "mobile health" OR MW teletherapy OR MW telediagnosis OR MW telepsychology OR MW tele-psychology OR MW telerehabilitation OR MW tele-rehabilitation OR MW e-rehabilitation OR MW "remote rehabilitation" OR MW "virtual rehabilitation" OR MW home-based OR ((MW web OR MW online OR MW internet OR MW digital) N2 (MW intervention OR MW treatment OR MW therapy OR MW based)) OR (MW mobile N2 (MW app OR MW application)) OR MW videoconferenc* OR MW "video conferenc*") |
| S4 | ((TI combin* OR AB combin*) OR (TI integrat* OR AB integrat*) OR (TI "'in conjuction'" OR AB "'in conjuction'") OR (TI hybrid OR AB hybrid) OR (TI blend* OR AB blend*) OR (TI addition* OR AB addition*) OR (TI adjunct* OR AB adjunct*) OR (TI complement* OR AB complement*)) |
| S5 | ((TI "usual care" OR AB "usual care") OR (TI "care as usual" OR AB "care as usual") OR (TI standard OR AB standard) OR (TI conventional OR AB conventional) OR (TI traditional OR AB traditional) OR (TI in-person OR AB in-person) OR (TI "face to face" OR AB "face to face") OR (TI in-hospital OR AB in-hospital) OR (TI hospital-based OR AB hospital-based) OR (TI inpatient OR AB inpatient) OR (TI in-patient OR AB in-patient) OR (TI in-clinic OR AB in-clinic) OR (TI clinic-based OR AB clinic-based) OR (TI "center based" OR AB "center based") OR (TI "centre based" OR AB "centre based") OR (TI in-center OR AB in-center) OR (TI in-centre OR AB in-centre) OR (TI onsite OR AB onsite) OR (TI on-site OR AB on-site) OR (TI outpatient OR AB outpatient) OR (TI out-patient OR AB out-patient)) |
| S6 | S1 AND S2 |
| S7 | S1 AND S3 AND S4 AND S5 |
| S8 | S6 OR S7 |
| S9 | (MH "Qualitative Studies+") OR (TI protocol OR TI qualitative) |
| S10 | S8 NOT S9 |

**Web of Science**

WoS search strategy

| #1 | (TI=("allied health" OR "paramedical personnel" OR rehabilitation OR neurorehabilitation OR telerehabilitation OR "tele-rehabilitation" OR "e-rehabilitation" OR "remote rehabilitation" OR "virtual rehabilitation" OR "functional assessment" OR "occupational therap*" OR physiotherap* OR kinesiotherapy OR exercise OR "muscle training" OR "speech language patholog*" OR "speech therap*" OR "language therap*" OR "music therap*" OR "art therap*" OR dietitian* OR dietetics OR "diet therapy" OR "dietary intervention" OR audiolog* OR hearing OR psychologist* OR therapist* OR psychotherap* OR telepsychology OR "tele-psychology" OR "social work*" OR "family therap*" OR "cognitive behavioral therapy") OR AK=("allied health" OR "paramedical personnel" OR rehabilitation OR neurorehabilitation OR telerehabilitation OR "tele-rehabilitation" OR "e-rehabilitation" OR "remote rehabilitation" OR "virtual rehabilitation" OR "functional assessment" OR "occupational therap*" OR physiotherap* OR kinesiotherapy OR exercise OR "muscle training" OR "speech language patholog*" OR "speech therap*" OR "language therap*" OR "music therap*" OR "art therap*" OR dietitian* OR dietetics OR "diet therapy" OR "dietary intervention" OR audiolog* OR hearing OR psychologist* OR therapist* OR psychotherap* OR telepsychology OR "tele-psychology" OR "social work*" OR "family therap*" OR "cognitive behavioral therapy") OR KP=("allied health" OR "paramedical personnel" OR rehabilitation OR neurorehabilitation OR telerehabilitation OR "tele-rehabilitation" OR "e-rehabilitation" OR "remote rehabilitation" OR "virtual rehabilitation" OR "functional assessment" OR "occupational therap*" OR physiotherap* OR kinesiotherapy OR exercise OR "muscle training" OR "speech language patholog*" OR "speech therap*" OR "language therap*" OR "music therap*" OR "art therap*" OR dietitian* OR dietetics OR "diet therapy" OR "dietary intervention" OR audiolog* OR hearing OR psychologist* OR therapist* OR psychotherap* OR telepsychology OR "tele-psychology" OR "social work*" OR "family therap*" OR "cognitive behavioral therapy")) |
| --- | --- |
| #2 | TI=(hybrid OR blended) OR AK=(hybrid OR blended) OR KP=(hybrid OR blended) |
| #3 | TI=(telehealth OR telemedicine OR e-health OR ehealth OR m-health OR mhealth OR  "mobile health" OR teletherapy OR telediagnosis OR telepsychology OR tele-psychology OR telerehabilitation OR tele-rehabilitation OR e-rehabilitation OR "remote rehabilitation" OR "virtual rehabilitation" OR home-based OR ((web OR online OR internet OR digital) NEAR/2 (intervention OR treatment OR therapy OR based )) OR (mobile NEAR/2 (app OR application)) OR videoconferenc* OR "video conferenc*") OR AK=(telehealth OR telemedicine OR e-health OR ehealth OR m-health OR mhealth OR  "mobile health" OR teletherapy OR telediagnosis OR telepsychology OR tele-psychology OR telerehabilitation OR tele-rehabilitation OR e-rehabilitation OR "remote rehabilitation" OR "virtual rehabilitation" OR home-based OR ((web OR online OR internet OR digital) NEAR/2 (intervention OR treatment OR therapy OR based )) OR (mobile NEAR/2 (app OR application)) OR videoconferenc* OR "video conferenc*") OR KP=(telehealth OR telemedicine OR e-health OR ehealth OR m-health OR mhealth OR  "mobile health" OR teletherapy OR telediagnosis OR telepsychology OR tele-psychology OR telerehabilitation OR tele-rehabilitation OR e-rehabilitation OR "remote rehabilitation" OR "virtual rehabilitation" OR home-based OR ((web OR online OR internet OR digital) NEAR/2 (intervention OR treatment OR therapy OR based )) OR (mobile NEAR/2 (app OR application)) OR videoconferenc* OR "video conferenc*") |
| #4 | (TI=(combin* OR integrat* OR "in conjuction" OR hybrid OR blend* OR addition* OR adjunct* OR complement*) OR AB=(combin* OR integrat* OR "in conjuction" OR hybrid OR blend* OR addition* OR adjunct* OR complement* )) |
| #5 | TI=("usual care" OR "care as usual" OR standard OR conventional OR traditional OR "in-person" OR "face to face" OR "in-hospital" OR "hospital-based" OR inpatient OR "in-patient" OR "in-clinic" OR "clinic-based" OR "center based" OR "centre based" OR "in-center" OR "in-centre" OR onsite OR "on-site" OR outpatient OR "out-patient") OR AB=("usual care" OR "care as usual" OR standard OR conventional OR traditional OR "in-person" OR "face to face" OR "in-hospital" OR "hospital-based" OR inpatient OR "in-patient" OR "in-clinic" OR "clinic-based" OR "center based" OR "centre based" OR "in-center" OR "in-centre" OR onsite OR "on-site" OR outpatient OR "out-patient") |
| #6 | #1 AND #2 |
| #7 | #1 AND #3 AND #4 AND #5 |
| #8 | #6 OR #7 |
| #9 | TI=(protocol OR qualitative) |
| #10 | #8 NOT #9 |

**PSYCHINFO**

Database: APA PsycInfo <1806 to January Week 5 2022>

Search Strategy:

--------------------------------------------------------------------------------

1 ('allied health' or 'paramedical personnel' or 'rehabilitation' or 'neurorehabilitation' or telerehabilitation or 'tele-rehabilitation' or 'e-rehabilitation' or 'remote rehabilitation' or 'virtual rehabilitation' or 'functional assessment' or 'occupational therap*' or 'physiotherap*' or kinesiotherapy or exercise or 'muscle training' or 'speech language patholog*' or 'speech therap*' or 'language therap*' or 'music therap*' or 'art therap*' or dietitian* or dietetics or 'diet therapy' or 'dietary intervention' or audiolog* or hearing or psychologist* or psychology or therapist* or psychotherap* or telepsychology or 'tele-psychology' or 'social work*' or 'family therap*' or 'cognitive behavioral therapy').ti,hw,id.

2 (hybrid or blended).ti,id.

3 (telehealth or telemedicine or "e-health" or ehealth or "m-health" or mhealth or "mobile health" or teletherapy or "e-therapy" or telediagnosis or telepsychology or "tele-psychology" or telerehabilitation or "tele-rehabilitation" or "e-rehabilitation" or "remote rehabilitation" or "virtual rehabilitation" or "home-based" or ((web or online or internet or digital) adj2 (intervention* or treatment* or therapy or based)) or (mobile adj2 (app or application)) or videoconferenc* or "video conferenc*").ti,hw,id.

4 (combin* or integrat* or 'in conjuction' or hybrid or blend* or addition* or adjunct* or complement*).ti,ab,id.

5 ("usual care" or "care as usual" or standard or conventional or traditional or "in-person" or "face to face" or "in-hospital" or "hospital-based" or inpatient or "in-patient" or "in-clinic" or "clinic-based" or "center based" or "centre based" or "in-center" or "in-centre" or onsite or "on-site" or outpatient or "out-patient").ti,ab,id.

6 1 and 2

7 1 and 3 and 4 and 5

8 6 or 7

9 (protocol or qualitative).ti.

10 exp Qualitative Methods/

11 9 or 10

12 8 not 11

13 limit 12 to all journals

***************************

**Cochrane CENTRAL**

Cochrane CENTRAL Search Strategy

| #1 | ("allied health" OR "paramedical personnel" OR rehabilitation OR neurorehabilitation OR telerehabilitation OR tele-rehabilitation OR e-rehabilitation OR "remote rehabilitation" OR "virtual rehabilitation" OR "functional assessment" OR ("occupational" NEXT therap*) OR physiotherap* OR kinesiotherapy OR exercise OR "muscle training" OR ("speech language" NEXT patholog*) OR ("speech" NEXT therap*) OR ("language" NEXT therap*) OR ("music" NEXT therap*) OR ("art" NEXT therap*) OR dietitian* OR dietetics OR "diet therapy" OR "dietary intervention" OR audiolog* OR hearing OR psychologist* OR psychology OR therapist* OR psychotherap* OR telepsychology OR tele-psychology OR ("social" NEXT work*) OR ("family" NEXT therap*) OR "cognitive behavioral therapy"):ti,kw |
| --- | --- |
| #2 | (hybrid OR blended):ti,kw |
| #3 | (telehealth OR telemedicine OR e-health OR ehealth OR m-health OR mhealth OR "mobile health" OR teletherapy OR telediagnosis OR telepsychology OR tele-psychology OR telerehabilitation OR tele-rehabilitation OR e-rehabilitation OR "remote rehabilitation" OR "virtual rehabilitation" OR home-based OR ((web  OR online OR internet OR digital) NEAR/2 (intervention OR treatment OR therapy OR based)) OR (mobile NEAR/2 (app OR application)) OR videoconferenc*  OR ("video" NEXT conferenc*)):ti,kw |
| #4 | (combin* OR integrat* OR "in conjuction" OR hybrid OR blend* OR addition* OR adjunct* OR complement*):ti,ab |
| #5 | ("usual care" OR "care as usual" OR standard OR conventional OR traditional OR "in-person" OR "face to face" OR "in-hospital" OR "hospital-based" OR inpatient OR "in-patient" OR "in-clinic" OR "clinic-based" OR "center based" OR "centre based" OR "in-center" OR "in-centre" OR onsite OR "on-site" OR outpatient OR "out-patient"):ti,ab,kw |
| #6 | #1 AND #2 |
| #7 | #1 AND #3 AND #4 AND #5 |
| #8 | #6 OR #7 in Trials |
